# Triglyceride-glucose body mass index and the risk of gestational diabetes mellitus: a population-based cohort study

**DOI:** 10.3389/fnut.2025.1657186

**Published:** 2025-12-31

**Authors:** Yezi Hu, Li Xu, Xiaojuan Wang, Zheng Zhou, Weiwei Yang, Yucui Yuan, Jiaxing Li, Shanhu Qiu, Yang Wang, Xuyi Wang, Pengbin Xia, Shaohua Wang, Hui Jin, Xiaoxia Zhu

**Affiliations:** 1 Department of Clinical Nutrition, Zhongda Hospital, School of Medicine, Southeast University, Nanjing, Jiangsu, China; 2 Chronic Disease Management Department, Community Health Service Center, Nanjing, Jiangsu, China; 3 Women's Health Department, Community Health Service Center, Nanjing, Jiangsu, China; 4 Research and Education Centre of General Practice, Zhongda Hospital Southeast University, Nanjing, Jiangsu, China; 5 Department of Endocrinology, Zhongda Hospital, School of Medicine, Southeast University, Nanjing, Jiangsu, China

**Keywords:** body composition, gestational diabetes mellitus, insulin resistance, pregnant women, triglyceride-glucose body mass index

## Abstract

**Background:**

The purpose of this analysis was to investigate the association between triglyceride-glucose body mass index (TyG-BMI) and gestational diabetes mellitus (GDM) in Chinese pregnant women.

**Methods:**

This observational study utilized a cohort of pregnant women who were screened for GDM using the oral glucose tolerance test (OGTT). The TyG-BMI was calculated using the formula: TyG index = ln (fasting triglycerides (mg/dL) × fasting glucose (mg/dL)/2). TyG-BMI = TyG × BMI. Statistical analyses were performed to assess the correlation between TyG-BMI and gestational diabetes mellitus incidence.

**Results:**

A total of 2,111 pregnant women were enrolled, out of which 281 (13.3%) women were diagnosed with gestational diabetes mellitus. Some differences existed between the TyG-BMI level groups with respect to various covariates (TG, BMI, age, TC, T-Bil, D-Bil, postpartum blood loss, vaginal birth, and GDM, *p* < 0.05). Compared to the lowest quartile of TyG-BMI, the third quartile of TyG-BMI is associated with the highest risk of developing gestational diabetes mellitus (OR: 1.8; 95%CI: 1.28 ~ 2.52). All models showed similar results. The stratified analyses were performed to examine whether the association between TyG-BMI and GDM was stable among different subgroups. None of the variables, including age (< 35 years and ≥35 years), premature delivery (yes or no), and vaginal birth (yes or no), significantly affected the association between TyG-BMI and GDM (all P for interaction > 0.05).

**Conclusion:**

A cohort study of Chinese pregnant women concluded that TyG-BMI might be a valuable index for identifying IR in patients at high risk of gestational diabetes mellitus. TyG-BMI could be recommended as part of routine surveillance during early pregnancy.

## 1 Introduction

Gestational diabetes mellitus (GDM) is characterized by impaired glucose metabolism, leading to elevated blood glucose levels during pregnancy (1, 2). However, its primary clinical significance lies in the series of complications it precipitates. Current evidence indicates a strong association between GDM and various adverse outcomes, including hypertensive disorders in pregnancy, increased rates of cesarean delivery, and long-term metabolic dysfunction in both mothers and offspring (3, 4). Of particular concern is that maternal GDM significantly elevates the risk of offspring developing obesity, impaired glucose tolerance, and type 2 diabetes mellitus during childhood and early adulthood (5), highlighting the disease’s transgenerational impact.

The current diagnostic paradigm relies on the oral glucose tolerance test performed between 24 and 28 weeks of gestation (6). This approach presents significant limitations: the relatively late diagnostic window leaves only approximately 12 weeks for intervention post-diagnosis, severely constraining the time available for effective management. Consequently, a critical challenge in current clinical practice is the establishment of earlier screening and risk assessment strategies to overcome the constraints of the current diagnostic timeline, thereby enabling early warning and proactive prevention and management of GDM.

Insulin resistance (IR) is a key factor in the development and progression of gestational diabetes mellitus (7). While the hyperinsulinemic-euglycemic clamp is considered the gold standard for measuring insulin sensitivity, its practical use is limited due to its labor-intensive and resource-heavy requirements (8). In contrast, the triglyceride-glucose (TyG) index has become a reliable surrogate marker for identifying IR, supported by multiple studies (9–11). Additionally, body mass index (BMI) is a well-recognized factor that influences IR (12). Recent research has investigated the connection between the TyG-BMI index and type 2 diabetes mellitus (T2DM), showing that higher TyG-BMI levels are significantly linked to an increased risk of developing T2DM. Elevated TyG-BMI values are generally associated with higher blood glucose levels and greater insulin resistance (13).

The TyG-BMI, a novel marker derived from fasting triglycerides, glucose levels, and body mass index, has been suggested as a potential indicator of insulin resistance and metabolic dysfunction. To the best of our knowledge, there are currently no articles available that examine the relationship between TyG-BMI and gestational diabetes mellitus. This study aims to investigate the association between TyG-BMI and gestational diabetes mellitus, offering insights into possible prevention strategies.

## 2 Methods

### 2.1 Study design and participants

**Figure 1:**
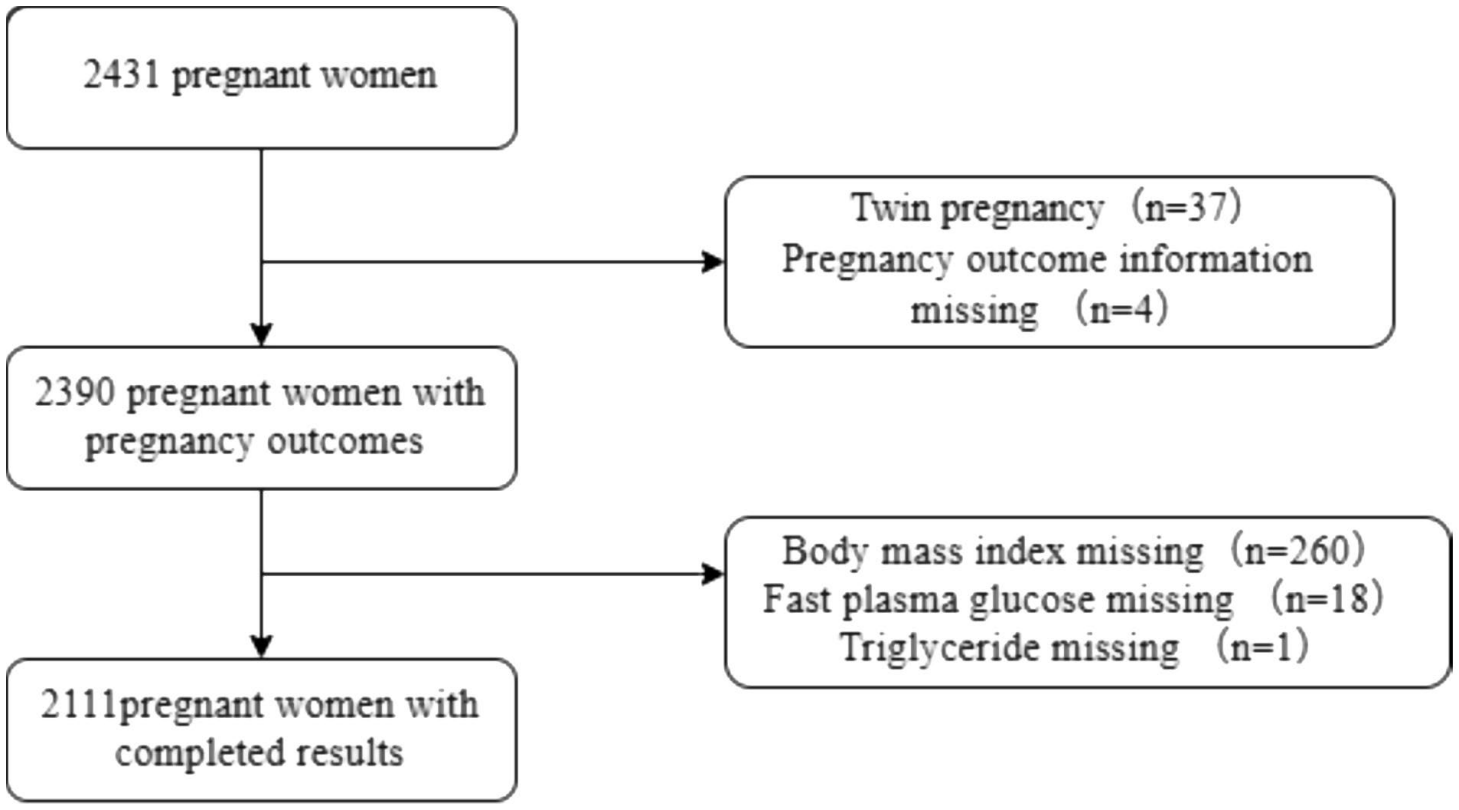
Flowchart of participant selection.


We retrospectively collected data on pregnant women who registered for antenatal care in the Yanjiang community from July 2022 to July 2023. Women with pre-pregnancy diabetes, severe liver or kidney disease, serious cerebrovascular disease, serious psychiatric disorders, intellectual disability, and malignant tumors were excluded. Initially, 2,431 pregnant women were recruited. Among them, 37 were excluded due to twin pregnancies, 4 did not have pregnancy outcomes, 260 were missing BMI data, 18 were missing FPG data, and 1 was missing TG data. Ultimately, 2,111 pregnant women were included in the study. The flowchart displaying patient selection is presented in Figure 1. Since BMI, FPG, and TG are essential raw data for calculating TyG-BMI and cannot be imputed, we directly excluded cases with missing data during collection to ensure the accuracy of the research findings (Figure 1). The sample size was determined based on the following calculation: Assuming a GDM prevalence of approximately 15% in China, a patient loss-to-follow-up rate of 10%, *α* = 0.05, and a margin of error (d) of 0.03, the required sample size was calculated to be 544. Although the calculated minimum sample size was 544, we enrolled a larger cohort of 2,111 participants. This decision was made to enhance the statistical power and reliability of our findings, especially considering that this is a single-center study. A larger sample helps to provide more robust estimates and strengthens the generalizability of our conclusions within the studied population.

$$n=\frac{Z_{1-\alpha /2}^{2}\times p\times (1-p)}{d^{2}}$$

The study was conducted in accordance with the principles outlined in the Declaration of Helsinki and received approval from the Ethics Review Board of Zhongda Hospital, Southeast University (Ethical Application Ref: 2023ZDSYLL169-122 P01; Ethical Application Date: 26-5-2023). This retrospective observational analysis was explicitly included in the original ethics-approved protocol for an interventional study. Due to the retrospective nature of the research and the use of anonymized patient data, the requirement for informed consent was waived.

### 2.2 Measurements and definitions

Fasting plasma glucose (FPG), triglyceride (TG), total cholesterol (TC), Low-density lipoprotein cholesterol (LDL-C), high-density lipoprotein cholesterol (HDL-C), hemoglobin (Hb), uric acid (UA), creatinine (Cr), total bilirubin (T-Bil), direct bilirubin (D-Bil), indirect bilirubin (I-Bil), free triiodothyronine (FT3), free thyroxine (FT4), thyrotropin (TSH), total protein (TP), albumin (Alb), globin (Glb), glycated hemoglobin A1c (HbA1c), hemoglobin (Hb), lactate dehydrogenase (LDH), alanine aminotransferase (ALT), and aspartate aminotransferase (AST) levels were tested after more than 8 h of fasting. The TyG index was calculated as previously described. The triglyceride-glucose (TyG) index, computed as ln[fasting triglycerides (mg/dl) × fasting blood glucose (mg/dl)/2] (9). Height was measured to the nearest 0.1 cm with a portable stadiometer. Weight was measured in an upright position to the nearest 0.1 kg with a calibrated scale.

Body mass index (BMI) was calculated as weight (kg)/height^2^ (m^2^) (14). TyG-BMI = TyG × BMI (13). The BMI values were confirmed to be pre-pregnancy measurements. The fasting blood glucose and fasting triglyceride levels required for calculating the TyG-BMI index were obtained from initial blood tests performed when pregnant women established their prenatal records at community hospitals between 9 and 13 weeks of gestation. The diagnosis of gestational diabetes mellitus was based on the IADPSG/WHO criteria, which stipulate that one or more glucose values from a 75-g OGTT must meet or exceed the following thresholds: fasting plasma glucose (FPG) of 5.1 mmol/L, 1-h plasma glucose (PG) of 10.0 mmol/L, and 2-h PG of 8.5 mmol/L (15).

### 2.3 Statistical analysis

Descriptive analysis was performed for categorical variables according to TyG-BMI quartiles (<168.452; 168.452–181.44; 181.44–199.064; ≥199.064) using the Kruskal–Wallis test or one-way analysis. For categorical variables, baseline characteristic data were presented as proportions (%) and compared using chi-square tests. Descriptive statistics are shown as mean ± standard deviation or medians (25th percentile, 75th percentile) for continuous variables with regard to the normality of distribution, while frequency and percentage were used for categorical variables.

We constructed three multivariate logistic models to assess the independent association between TyG-BMI and gestational diabetes mellitus: Model 1 was a crude model (adjusted); Model 2 was adjusted for age+ FPG + TG + BMI; and Model 3 was additionally adjusted for AST + ALT +TC + T-Bil + D-Bil + Cr + HDL. A test for linear trends was conducted using quartiles of the exposure variable as a continuous variable by assigning the median values of the quartiles to the variable.

All analyses were performed with the statistical software packages R (http://www.R-project.org, The R Foundation) and Free Statistics software versions 2.0. A two-sided *p*-value of <0.05 was considered to be statistically significant.

## 3 Results

### 3.1 Baseline characteristics

The baseline characteristics of study participants, categorized according to their TyG-BMI levels, are presented in Table 1. This study included a total of 2,111 pregnant women, among whom 281 individuals, accounting for 13.3% of the total sample, were diagnosed with gestational diabetes mellitus. Significant differences were observed between the groups stratified by TyG-BMI levels regarding several covariates. These covariates included triglycerides (TG), body mass index (BMI), age, total cholesterol (TC), total bilirubin (T-Bil), direct bilirubin (D-Bil), postpartum blood loss, vaginal delivery, and the prevalence of GDM (all *p*-values < 0.05). Such differences highlight the potential influence of TyG-BMI on these variables and their possible relevance to the development of gestational diabetes mellitus.

**Table 1:** Clinical characteristics at baseline in the four categories of TyG-BMI index.

Variables	Overall	TyG-BMI quartiles				*p*
	Total (*n* = 2,111)	Q1 (*n* = 528)	Q2 (*n* = 527)	Q3 (*n* = 528)	Q4 (*n* = 528)	
FPG (mg/dL)	86.3 ± 7.4	86.1 ± 6.4	86.4 ± 7.4	86.9 ± 9.1	85.9 ± 6.3	0.164
TG (mg/dL)	128.2 ± 53.8	110.5 ± 42.0	128.4 ± 52.7	129.5 ± 51.9	144.6 ± 61.5	< 0.001
BMI (kg/m^2^)	21.8 ± 2.9	18.9 ± 1.1	20.6 ± 1.0	22.2 ± 1.1	25.6 ± 2.6	< 0.001
TyG-BMI	186.2 ± 25.8	158.6 ± 7.8	175.4 ± 3.5	189.7 ± 5.1	221.1 ± 21.5	< 0.001
Age (year)	30.1 ± 3.5	31.6 ± 3.9	30.2 ± 3.4	29.4 ± 3.1	29.3 ± 3.0	< 0.001
AST (U/L)	17.1 ± 8.5	16.8 ± 8.7	17.0 ± 7.5	17.5 ± 10.0	17.3 ± 7.7	0.663
ALT (U/L)	13.0 (9.0, 20.0)	12.0 (9.0, 19.0)	13.0 (9.0, 20.0)	12.0 (9.0, 20.0)	13.0 (9.0, 20.0)	0.908
LDH (U/L)	150.4 ± 20.8	150.4 ± 22.0	151.4 ± 21.5	149.5 ± 20.4	150.3 ± 19.2	0.649
HDL -C (mmol/L)	1.7 ± 0.3	1.7 ± 0.4	1.7 ± 0.3	1.7 ± 0.3	1.7 ± 0.3	0.307
LDL-C (mmol/L)	2.3 ± 0.6	2.2 ± 0.7	2.3 ± 0.6	2.3 ± 0.5	2.3 ± 0.6	0.281
UA(mmol/L)	209.5 ± 47.4	207.2 ± 47.4	209.8 ± 49.0	211.7 ± 48.5	209.5 ± 44.8	0.49
TC (mmol/L)	4.5 ± 0.8	4.4 ± 0.8	4.5 ± 0.7	4.4 ± 0.7	4.5 ± 0.8	< 0.001
T-Bil (umol/L)	7.5 ± 3.2	7.8 ± 3.3	7.5 ± 3.2	7.4 ± 3.1	7.2 ± 3.1	0.014
D-Bil (umol/L)	3.3 ± 1.2	3.5 ± 1.3	3.3 ± 1.2	3.3 ± 1.2	3.2 ± 1.2	0.001
I-Bil (umol/L)	4.1 ± 2.1	4.3 ± 2.1	4.2 ± 2.1	4.1 ± 2.0	4.0 ± 2.0	0.078
FT3 (pg/mL)	4.7 ± 0.9	4.7 ± 0.8	4.7 ± 1.1	4.8 ± 0.9	4.7 ± 1.0	0.281
FT4 (pg/mL)	18.2 ± 3.4	18.3 ± 3.2	18.2 ± 3.6	18.4 ± 3.2	17.9 ± 3.5	0.132
TSH (uIU/mL)	1.4 ± 1.2	1.4 ± 1.2	1.5 ± 1.4	1.4 ± 1.2	1.3 ± 0.9	0.251
TP (g/L)	70.3 ± 3.6	70.3 ± 3.6	70.3 ± 3.6	70.5 ± 3.4	70.1 ± 3.7	0.303
Alb (g/L)	44.4 ± 2.6	44.3 ± 2.5	44.2 ± 2.7	44.6 ± 2.5	44.4 ± 2.6	0.115
Glb (g/L)	25.9 ± 3.2	26.0 ± 3.2	26.1 ± 3.3	25.9 ± 3.2	25.7 ± 3.0	0.165
HbA1c (%)	5.3 ± 0.3	5.3 ± 0.3	5.3 ± 0.3	5.3 ± 0.4	5.3 ± 0.3	0.17
Cr (umol/L)	48.3 ± 6.4	48.4 ± 5.7	48.4 ± 6.6	48.5 ± 6.5	48.0 ± 6.6	0.649
Hb (g/L)	128.6 ± 10.1	129.0 ± 9.7	127.9 ± 10.6	128.7 ± 10.4	128.9 ± 9.6	0.268
Postpartum blood loss (ml)	239.2 ± 265.3	222.6 ± 237.9	251.9 ± 354.9	211.7 ± 223.0	270.4 ± 216.4	0.001
Birth Weight (g)	3341.8 ± 450.2	3326.4 ± 460.0	3360.3 ± 429.1	3327.9 ± 460.1	3352.4 ± 451.2	0.511
ABO blood groups *n* (%)						0.729
AB group	178 (8.5)	42 (8)	50 (9.6)	41 (7.8)	45 (8.6)	
A group	663 (31.5)	162 (30.7)	164 (31.4)	182 (34.7)	155 (29.5)	
B group	587 (27.9)	155 (29.4)	142 (27.2)	133 (25.3)	157 (29.8)	
O group	674 (32.1)	169 (32)	167 (31.9)	169 (32.2)	169 (32.1)	
AB group	178 (8.5)	42 (8)	50 (9.6)	41 (7.8)	45 (8.6)	
Vaginal birth *n* (%)						< 0.001
No	883 (41.8)	258 (48.9)	268 (50.9)	188 (35.6)	169 (32)	
Yes	1,228 (58.2)	270 (51.1)	259 (49.1)	340 (64.4)	359 (68)	
Premature delivery *n* (%)						0.454
No	2019 (95.6)	500 (94.7)	509 (96.6)	503 (95.3)	507 (96)	
Yes	92 (4.4)	28 (5.3)	18 (3.4)	25 (4.7)	21 (4)	
Macrosomia *n* (%)						0.988
No	1971 (93.4)	494 (93.6)	491 (93.2)	492 (93.2)	494 (93.6)	
Yes	140 (6.6)	34 (6.4)	36 (6.8)	36 (6.8)	34 (6.4)	
GDM *n* (%)						< 0.001
No	1830 (86.7)	454 (86)	458 (86.9)	414 (78.4)	504 (95.5)	
Yes	281 (13.3)	74 (14)	69 (13.1)	114 (21.6)	24 (4.5)	

TyG-BMI, triglyceride glucose-body mass index; triglyceride, glucose-body mass index; FPG, fasting plasma glucose; TG, triglyceride; BMI, body mass index; AST, aspartate aminotransferase; ALT, alanine aminotransferase; LDH, lactate dehydrogenase; HDL -C, high density lipoprotein cholesterol; LDL-C, low density lipoprotein cholesterol; UA, uric acid; TC, total cholesterol; T-Bil, Total bilirubin; D-Bil, direct bilirubin; I-Bil, indirect bilirubin; FT3, free triiodothyronine; FT4, free thyroxine; TSH, thyrotropin; TP, total protein; Alb, albumin; Glb, globin; HbA1c, glycated hemoglobin A1c; Cr, creatinine; Hb, hemoglobin; GDM, gestational diabetes mellitus.

### 3.2 TyG-BMI and risk of incident GDM

The association between TyG-BMI levels and the risk of developing gestational diabetes mellitus was evaluated using logistic regression analysis, with the results summarized in Table 2. When compared to the lowest quartile of TyG-BMI, the third quartile demonstrated the strongest association with an increased risk of developing GDM. Specifically, the odds ratio (OR) for the third quartile was 1.8, with a 95% confidence interval (CI) of 1.28 to 2.52. This indicates a significantly higher likelihood of gestational diabetes mellitus occurrence in this group. Similar trends were observed across all regression models, reinforcing the robustness of the findings. While the second quartile and the highest quartile of TyG-BMI also showed associations with the risk of gestational diabetes mellitus, the results were not statistically significant, as indicated by *p*-values greater than 0.05. This suggests that the relationship between TyG-BMI and GDM may vary depending on the specific quartile, with the third quartile showing the strongest effect.

**Table 2:** Results of multivariate analysis between TyG-BMI index and GDM.

Variable	*n* total	Event *n*%	Crude OR (95%CI)	Crude *p*-value	Model 1 adj. OR (95%CI)	Model 1 *p*-value	Model 2 OR (95%CI)	Model 2 adj. *p*-value
TyG-BMI Q1	528	74 (14)	1 (Ref)		1(Ref)		1(Ref)	
TyG-BMI Q2	527	69 (13.1)	0.92 (0.65 ~ 1.32)	0.662	0.97 (0.68 ~ 1.38)	0.859	0.94 (0.65 ~ 1.34)	0.719
TyG-BMI Q3	528	114 (21.6)	1.69 (1.22 ~ 2.33)	0.001	1.82 (1.31 ~ 2.54)	<0.001	1.8 (1.28 ~ 2.52)	0.001
TyG-BMI Q4	528	24 (4.5)	0.29 (0.18 ~ 0.47)	<0.001	0.32 (0.19 ~ 0.51)	<0.001	0.3 (0.18 ~ 0.49)	<0.001
P for trend	2,111			0.003		0.009		0.005

Crude Model: unadjusted.

Model 1: adjust for age.

Model 2: adjust for age+AST+ALT +TC +T-Bil +D-Bil+ Cr+ HDL.

TyG-BMI, triglyceride glucose-body mass index; GDM, gestational diabetes mellitus; OR, odd ratio; CI, confidence interval; Ref: reference.

### 3.3 Stratified analyses

To ensure the consistency of the observed association between TyG-BMI and GDM across different subgroups, stratified analyses were conducted. These analyses examined whether factors such as maternal age (<35 years versus ≥35 years), the presence or absence of premature delivery, and the mode of delivery (vaginal birth or not) influenced the relationship between TyG-BMI and GDM. The results, as presented in Table 3, indicate that none of these variables significantly modified the association, as all *p*-values for interaction were greater than 0.05. This suggests that the relationship between TyG-BMI and GDM was stable and consistent across the subgroups analyzed.

**Table 3:** Stratification analysis on the association between TyG-BMI and GDM.

Subgroup	Variable	*N*	*N* event %	Adjusted OR (95%CI)	Adjusted P_value	P for interaction
Age < 35						0.446
	TyG-BMI Q1	372	45 (12.1)	1 (Ref)		
	TyG-BMI Q2	481	61 (12.7)	1.06 (0.7 ~ 1.59)	0.797	
	TyG-BMI Q3	514	110 (21.4)	1.98 (1.36 ~ 2.88)	<0.001	
	TyG-BMI Q4	510	24 (4.7)	0.36 (0.21 ~ 0.6)	<0.001	
	P for trend				0.024	
Age ≥ 35						
	TyG-BMI Q1	156	29 (18.6)	1 (Ref)		
	TyG-BMI Q2	46	8 (17.4)	0.92 (0.39 ~ 2.18)	0.854	
	TyG-BMI Q3	14	4 (28.6)	1.75 (0.51 ~ 5.98)	0.371	
	TyG-BMI Q4	18	0 (0)	0 (0 ~ Inf)	0.986	
	P for trend				0.23	
Premature delivery (No)						0.332
	TyG-BMI Q1	500	69 (13.8)	1(Ref)		
	TyG-BMI Q2	509	67 (13.2)	0.95 (0.66 ~ 1.36)	0.767	
	TyG-BMI Q3	503	107 (21.3)	1.69 (1.21 ~ 2.35)	0.002	
	TyG-BMI Q4	507	23 (4.5)	0.3 (0.18 ~ 0.48)	<0.001	
	P for trend				0.003	
Premature delivery (Yes)						
	TyG-BMI Q1	28	5 (17.9)	1 (Ref)		
	TyG-BMI Q2	18	2 (11.1)	0.58 (0.1 ~ 3.34)	0.538	
	TyG-BMI Q3	25	7 (28)	1.79 (0.49 ~ 6.58)	0.382	
	TyG-BMI Q4	21	1 (4.8)	0.23 (0.02 ~ 2.14)	0.196	
	P for trend				0.562	
Vaginal birth (No)						0.329
	TyG-BMI Q1	258	44 (17.1)	1 (Ref)		
	TyG-BMI Q2	268	42 (15.7)	0.9 (0.57 ~ 1.44)	0.668	
	TyG-BMI Q3	188	40 (21.3)	1.31 (0.82 ~ 2.12)	0.261	
	TyG-BMI Q4	169	8 (4.7)	0.24 (0.11 ~ 0.53)	<0.001	
	P for trend				0.014	
Vaginal birth (Yes)						
	TyG-BMI Q1	359	30 (11.1)	1 (Ref)		
	TyG-BMI Q2	270	27 (10.4)	0.93 (0.54 ~ 1.61)	0.799	
	TyG-BMI Q3	259	74 (21.8)	2.23 (1.41 ~ 3.52)	0.001	
	TyG-BMI Q4	340	16 (4.5)	0.37 (0.2 ~ 0.7)	0.002	
	P for trend				0.151	

In the subgroup aged 35 years and older, the number of GDM events is none, resulting in an abnormal OR value.

TyG-BMI, triglyceride glucose-body mass index; GDM, gestational diabetes mellitus; OR, odds ratio; CI, confidence interval; Q: quartile; Ref: reference.

## 4 Discussion

To the best of our knowledge, our current large population-based cohort study consistently demonstrates a noteworthy association between TyG-BMI and the risk level of gestational diabetes mellitus for the first time. Compared to the lowest quartile of TyG-BMI, the third quartile is associated with the highest risk of developing gestational diabetes mellitus. This association remains significant even when accounting for various clinical subgroups. These findings have important clinical implications for the management and prevention of gestational diabetes mellitus.

Currently, research on TyG-BMI is predominantly concentrated in populations with established cardiometabolic and renal conditions, including cardiovascular diseases, chronic kidney disease, and other metabolic disorders (16–18). While the triglyceride-glucose index (TyG) itself is recognized as a promising marker in preventive cardiology and cardiometabolic medicine (19), emerging evidence suggests that TyG-BMI may offer enhanced prognostic utility. For instance, Chen et al. demonstrated that in patients with chronic kidney disease, TyG-BMI exhibits stronger prognostic diagnostic value than TyG alone, and subgroup analyses indicated it is more significantly associated with all-cause and cardiovascular mortality in elderly patients (20). Similarly, in patients undergoing peritoneal dialysis, elevated baseline TyG-BMI was independently associated with increased risks of CVD and all-cause mortality (21).

However, the relationship appears complex and context-dependent. Analyses from the NHANES study revealed a U-shaped association between TyG-BMI and all-cause or CVD mortality among diabetic patients (22), indicating non-linearity that complicates its clinical interpretation. Furthermore, while higher TyG-BMI levels have been linked to conditions such as nephrolithiasis (23) and hyperuricemia in certain populations, its predictive value is not consistent across all clinical scenarios. Notably, one study found that neither TyG nor TyG-BMI served as an independent risk factor for major adverse cardiovascular and cerebrovascular events during 12-month follow-up in patients with myocardial infarction (24, 25).

IR plays an important role in the development of various metabolic diseases, such as type 2 diabetes, dyslipidemia, and obesity (26–28). Many studies have also shown that TyG or TyG-BMI are closely associated with IR (29–32).

There is still no definitive consensus on whether TyG-BMI or the TyG index serves as a better proxy for insulin resistance; rather, it seems that the suitability of each indicator may differ across various study populations. In our investigation involving a cohort of pregnant women, we explored the relationship between TyG-BMI and gestational diabetes mellitus, finding that TyG-BMI shows a stronger association with gestational diabetes mellitus. Notably, a higher TyG-BMI does not consistently correlate with an increased risk of gestational diabetes mellitus. Our findings indicate that, compared to the lowest quartile of TyG-BMI, the third quartile is associated with the highest risk of developing gestational diabetes mellitus. The generalizability of these findings is limited to the specific community included in our study. Further investigations involving diverse populations and ethnic groups are thus warranted to validate and extend our observations.

In clinical practice, there is often a focus on populations at either end of the TyG-BMI spectrum, frequently neglecting those in the intermediate range. Our research highlights the importance of considering this middle group, as they may also be at significant risk. One possible explanation for this observation is that the TyG-BMI metric includes BMI, which does not differentiate between muscle and fat mass, thereby partially explaining the obesity paradox (33). This may partially explain why the highest quartile (Q4) of TyG-BMI demonstrated a lower risk of GDM compared to Q1.

Gestational diabetes mellitus is a common metabolic complication during pregnancy, characterized by a complex pathogenesis involving multiple biological principles and mechanisms. Recent studies have demonstrated that placental metabolic changes, obesity, and the TyG-BMI index are closely associated with the pathogenesis of GDM. The placenta plays a particularly important role in GDM, as it serves not only as the central site for nutrient exchange between the mother and fetus but also participates in the regulation of various metabolic and endocrine functions (34, 35). First, DNA methylation changes in the placenta are considered one of the key factors in the pathogenesis of GDM. Studies have shown that placentas from GDM patients exhibit significantly elevated levels of DNA methylation, which may affect the expression of placenta-related genes and thereby disrupt normal placental function (36, 37). Additionally, the upregulation of inflammatory factors in the placenta, such as increased expression of IL-15, has been closely linked to the development of GDM. These factors alter the biological behavior of trophoblast cells by activating the JAK/STAT signaling pathway, thereby contributing to placental pathological changes (38). Second, the relationship between obesity and GDM has gained widespread attention. Obesity not only increases the risk of GDM but also exacerbates insulin resistance and chronic inflammation by influencing adipokines (e.g., leptin and adiponectin) secreted by adipose tissue, thereby promoting the onset of GDM (39, 40). Furthermore, variations in the FTO gene, which are associated with obesity, may exacerbate the pathological process of GDM by affecting adipokine levels (41). Finally, the TyG-BMI index, as a novel metabolic indicator, effectively assesses insulin resistance and lipid metabolism status. Research has shown that the TyG-BMI index is closely related to the occurrence of GDM, and its elevation may reflect aggravated insulin resistance and dysregulated lipid metabolism (42, 43). Compared to traditional surrogate indices for insulin resistance, the TyG-BMI index offers the advantages of being readily available and cost-effective, as it does not require additional measurements such as C-peptide or insulin levels, thereby reducing the financial burden on patients. Moreover, as a composite index, TyG-BMI integrates the influences of pre-pregnancy BMI, early-pregnancy lipid profiles, and fasting blood glucose, enabling it to more accurately reflect the level of insulin resistance in pregnant women than any single indicator alone.

Insulin resistance can take years to progress to type 2 diabetes (44), and experiencing insulin resistance during pregnancy, along with a hyperglycemic environment for the fetus, may elevate the risk of developing diabetes for both the mother and child later in life. Therefore, early identification of insulin resistance during pregnancy is especially important. While populations with very high or very low TyG-BMI often receive more attention, our study suggests that the intermediate range of TyG-BMI also warrants consideration. We hope that our research provides valuable insights into improving maternal and infant health outcomes.

Although we are the first to examine the relationship between TyG-BMI and GDM, we do have some limitations. First, our analysis is based on an observational cohort study. Second, while participants with specific health conditions were excluded, it may be challenging to guarantee complete certainty regarding the absence of underlying diseases that could affect blood glucose, lipid levels, or insulin secretion. Additionally, only baseline TyG-BMI data were analyzed, and we did not obtain the raw data on parity of the pregnant women either. Due to limitations in data collection inherent to the retrospective study design, detailed glucose distributions from the OGTT results and gestational weight gain data could not be obtained. In this study, cases with missing data for BMI, FPG, or TG were excluded from the analysis, as these parameters were essential for calculating the primary exposure variable, TyG-BMI. Consequently, this approach may introduce potential for selection bias. However, our large sample size (*n* = 2,111) is expected to enhance the representativeness of the included cohort and may, to some extent, mitigate the impact of this limitation. It is worth noting that in the subgroup of women aged 35 years and older, the number of gestational diabetes mellitus cases was extremely low. This scarcity of events resulted in an abnormal odds ratio, limiting the reliability of the findings for this particular group. Therefore, longitudinal cohort studies are needed to investigate whether the association between TyG-BMI and GDM changes over time.

## 5 Conclusion

A cohort study of Chinese pregnant women concluded that TyG-BMI might be a valuable index for identifying IR in patients at high risk of gestational diabetes mellitus. TyG-BMI could be recommended as part of routine surveillance during early pregnancy.

## Data Availability

As this retrospective analysis is a component of an ongoing interventional study, the datasets generated and analyzed are not currently publicly available. The full data will be shared upon completion of the primary study. Requests to access the datasets should be directed to YH, hyz1932sun@163.com.
